# Childhood leukaemia and socioeconomic status in England and Wales 1976–2005: evidence of higher incidence in relatively affluent communities persists over time

**DOI:** 10.1038/bjc.2011.415

**Published:** 2011-10-25

**Authors:** M E Kroll, C A Stiller, M F G Murphy, L M Carpenter

**Affiliations:** 1 Childhood Cancer Research Group, University of Oxford, Richards Building, Oxford OX3 7LG, UK; 2 Cancer Epidemiology Unit, University of Oxford, Richard Doll Building, Oxford OX3 7LF, UK; 3 Department of Public Health, University of Oxford, Rosemary Rue Building, Oxford OX3 7LF, UK; 4 Nuffield College, University of Oxford, New Road, Oxford OX1 1NF, UK

**Keywords:** childhood leukaemia, socioeconomic status, deprivation, affluence, delayed infection

## Abstract

**Background:**

Record-based studies have generally reported association of higher childhood leukaemia incidence with higher socioeconomic status (SES), but recent findings are less consistent.

**Methods:**

We examined records from the National Registry of Childhood Tumours for evidence of this association in England and Wales during 1976–2005. All eligible leukaemia registrations (*N*=11940) were grouped by year of diagnosis in decades centred on census years 1981, 1991 and 2001 (*N*=3748, 3922, 4270, respectively). Using data from the census appropriate to the decade, SES for each case was measured by the child-population-weighted quintile of the Carstairs deprivation index of the census ward containing the address at diagnosis.

**Results:**

In each decade, the age-standardised leukaemia rate in the poorest quintile was ∼90% of the rate in the most affluent. Using Poisson regression, the age-adjusted rate ratio per quintile decrease in SES was 0.96 (95% confidence interval 0.94–0.98; *P*<0.001 for trend) in 1976–1985, 0.97 (0.95–0.99; *P*=0.008) in 1986–1995 and 0.97 (0.95–0.99; *P*=0.009) in 1996–2005. Similar association was evident for lymphoid leukaemia, the major subgroup (*N*=9588 in total), but not for acute myeloid (*N*=1868) or other/unspecified leukaemia (*N*=484).

**Conclusion:**

Reported childhood leukaemia incidence in England and Wales continues to be higher in relatively affluent communities. Possible explanations include under-diagnosis of leukaemia in children from poorer communities, and/or association of higher SES with hypothesised risk factors, such as population mixing and delayed exposure to infection.

Record-based studies have reported higher childhood leukaemia incidence in populations with higher socioeconomic status (SES), most evidently for lymphoid leukaemia, the largest subtype (Doll, 1989). This subject is of particular interest because affluence may be associated with various hypothesised risk factors for childhood leukaemia, including population mixing and delayed exposure to infection (McNally and Eden, 2004).

A recent systematic review of the evidence concerning association between childhood leukaemia and SES identified 47 eligible studies published during 1945–2002, and reported that results were heterogeneous over time (Poole *et al*, 2006). In general, earlier studies suggested that risks were elevated in relatively affluent children, whereas later studies suggested that risks were reduced in relatively affluent children (inverse association). Potential explanations for this heterogeneity included variation in study design: most of the earlier studies were record-based (incidence studies using registry data, or case–control studies that did not entail subject participation), whereas most of the later studies were case–control studies involving interviews or questionnaires. The inverse associations, when found, did not appear to vary with leukaemia subtype, and might be due to control selection bias (Poole *et al*, 2006). A later review reported heterogeneity in subsequent studies published up to 2008 (Adam *et al*, 2008).

The aim of the present study was to determine whether there was any change in the relationship between SES and recorded incidence of childhood leukaemia in England and Wales over the three decades from 1976 to 2005. The application of the same study design and method of analysis to each decade facilitates comparison of results between decades. To our knowledge, no previous national study of SES and childhood leukaemia has included cases from England and Wales diagnosed after 1996.

## Materials and methods

The National Registry of Childhood Tumours (NRCT) is a specialist registry recording childhood cancer diagnosed in England, Wales and Scotland since 1962 (Stiller, 2007; Kroll *et al*, 2011). For this study, cases consisted of NRCT registrations for leukaemia diagnosed from 1 January 1976 to 31 December 2005 in residents of England and Wales aged <15 years. Leukaemia was defined as diagnostic group I of the third edition of the International Classification of Childhood Cancer (ICCC3) (Steliarova-Foucher *et al*, 2005), excluding diseases classified as non-malignant in previous standard coding systems (ICDO-3 morphology codes 99313, 99503, 99603, 99613, 99623, 99803, 99823, 99833, 99843 and 99893). Following ICCC3, cases were subclassified as lymphoid (a), acute myeloid (b) or other/unspecified (c) to (e).

SES was assessed using a community-based measure of relative deprivation, the Carstairs index (Carstairs and Morris, 1989). This is the unweighted sum of z-scores for four variables derived from census data: the proportion of economically active adult males who are unemployed (1), and the proportions of residents living in households with overcrowding (one or more persons per room) (2), no car (3), and economically active head of household in low social class (IV and V) (4) (Morgan and Baker, 2006). Based on census wards in England and Wales, a separate Carstairs index was obtained for each of the three national censuses conducted during the study period: the 1981 index had been calculated for a previous study (Committee on Medical Aspects of Radiation in the Environment, 2006); the 1991 and 2001 indices were provided by the Census Dissemination Unit (ESRC Census Programme, 2010a) and the Office for National Statistics, respectively. There were ∼9000 wards within England and Wales at each census, although the boundaries and exact number of wards varied. Each set of census wards was grouped into quintile categories of the appropriate Carstairs index (deprivation categories), weighted to contain approximately equal numbers of children aged 0–14.

Cases were grouped by decade of diagnosis (1976–1985, 1986–1995 and 1996–2005). Using the ward boundaries and Carstairs index from the census taken at the central year of the decade of diagnosis, the SES of each case was measured by the deprivation category of the ward that contained the post code of the address at diagnosis (ESRC Census Programme, 2010b) (1=affluent, …, 5=deprived). For each decade, age-specific ward population counts from the appropriate census were multiplied by 10 and summed to obtain approximate child-years by deprivation category and age group (<1, 1–4, 5–9 and 10–14 completed years). Age-specific rates, and hence rates directly standardised by age group to a uniform population, were calculated by decade and deprivation category.

Poisson regression was used to model variation in rate by age group, decade and deprivation category (Rothman and Greenland, 1998; StataCorp, 2009). Models were compared by likelihood ratio tests. Rate ratios and trends by deprivation category were estimated separately within each decade (adjusting for age group), and for all three decades combined (adjusting for age group and decade). Terms representing interactions between age group and deprivation, and differences in deprivation trend between decades in the combined model, were tested and dropped from the final models because they were not statistically significant. Goodness of fit was tested by the deviance statistic and found to be adequate throughout. All statistical tests were two-sided, using the significance level of 5%.

## Results

**Figure 1:**
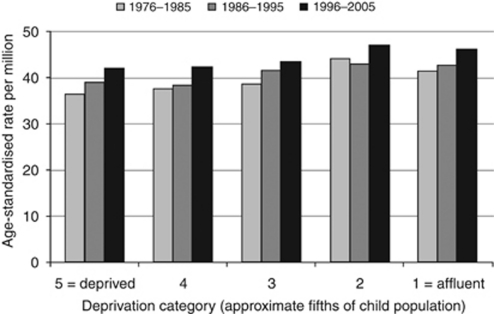
Recorded incidence of leukaemia in children under 15 years of age, England and Wales, 1976–2005. Age-standardised rates per million by decade and deprivation category.

In total, there were 11 974 registrations for leukaemia diagnosed during 1976–2005 in residents of England and Wales aged under 15 years. In all, 34 cases were excluded from the analysis because the postcode at diagnosis was not available. Of the remaining 11 940 cases, 80% (9588) were diagnosed as lymphoid leukaemia and 16% (1868) as acute myeloid leukaemia (Table 1). Leukaemia rates generally increased over time: in the most deprived fifth of children, for example, the age-standardised rate for total leukaemia was 36.5 per million in 1976–1985, 39.0 in 1986–1995 and 42.1 in 1996–2005 (Figure 1).

Within each of the three study decades, the age-standardised rate for total leukaemia was higher in relatively affluent children (Figure 1). In 1996–2005, the estimated rate ratio for the most deprived fifth of children relative to the most affluent fifth was 0.91 (95% confidence interval 0.83–1.01) (Table 2). Corresponding results were almost identical in 1986–1995, and only slightly different in 1976–1985 (0.89 (0.80–0.99)). In each decade, there was a highly significant log-linear trend in rate over categories of SES: the rate ratio per unit increase in deprivation quintile was 0.97 (0.95–0.99) in both 1996–2005 and 1986–1995, and 0.96 (0.94–0.98) in 1976–1985. Combining all three decades, the estimated overall trend was 0.97 (0.96–0.98) per quintile, and the rate ratio for the most deprived fifth of children relative to the most affluent fifth was 0.91 (0.86–0.96).

Patterns for the lymphoid subgroup alone were similar to those observed for total leukaemia, and the estimated trend for all three decades combined was 0.96 (0.95–0.98) per quintile of deprivation (Table 2). In contrast, for each of the other two subgroups (acute myeloid leukaemia and other/unspecified leukaemia), there was no statistically significant trend with deprivation in any decade, and rate ratios were close to unity for the three decades combined.

## Discussion

### Summary

During the three decades to 2005, recorded incidence of childhood leukaemia in England and Wales was persistently higher in relatively affluent communities. In each decade, the age-standardised leukaemia rate in the most deprived fifth of the child population was ∼90% of the rate in the most affluent fifth. There was little change in the strength of the association over time. Similar associations were evident for the lymphoid subgroup alone, but not for acute myeloid or other/unspecified leukaemia.

### Comparison with previous studies

These results are consistent with previous analyses of NRCT data for England and Wales up to 1995 (Committee on Medical Aspects of Radiation in the Environment, 2006; Stiller *et al*, 2008), and with the systematic review of studies published during 1945–2002 (Poole *et al*, 2006). Two subsequent record-based studies found similar associations: for total and lymphoid leukaemia under age 20 years in Canada during 1985–2001 (Borugian *et al*, 2005), and for lymphoid leukaemia under age 5 years in the United States during 1992–1998 (Adelman *et al*, 2007). Internationally, positive correlations have been reported between childhood lymphoid leukaemia rates and measures of affluence (Feltbower *et al*, 2004).

Some recent record-based studies have reported inverse or null associations. In Denmark, a registry-based case–control study (without subject participation) for cases diagnosed during 1976–1991 found elevated odds ratios for lymphoid leukaemia in children resident at birth in municipalities within the lowest decile of mean gross income, but no association for residence at diagnosis, or for individual SES measured by parental occupation (Raaschou-Nielsen *et al*, 2004). Within the United States during 2000–2005, an excess of leukaemia was reported in counties with medium and high poverty rates for age group 5–9 years, but not for the peak age group of 0–4 years, or other standard age groups under 20 years (Pan *et al*, 2010).

The United Kingdom Childhood Cancer Study (UKCCS) accrued 4430 cases of cancer diagnosed under age 15 years in England, Wales and Scotland between 1991 and 1996 (Smith *et al*, 2006). Matched controls were selected (without participation) for 3835 case children whose parents had been interviewed. When conditional logistic regression was applied to the 1460 matched sets of lymphoid leukaemia cases and controls, using a census-based deprivation score for address at diagnosis, a statistically significant decreasing trend was found with increasing deprivation. The estimated risk for the most deprived quintile category relative to the most affluent (0.76 with 95% confidence interval 0.61–0.95) was consistent with the results reported in a previous matched analysis of UKCCS data (Law *et al*, 2003), and compatible with the rate ratios reported here.

### Strengths and limitations

This is a large population-based study. No subject participation was involved. Throughout the study period, case records were routinely ascertained from defined multiple sources, with careful matching and validation. Sources included a register of patients seen by clinicians affiliated to the United Kingdom Children's Cancer Study Group (the organisation that co-ordinated paediatric oncology in Britain during 1977–2006, unrelated to the similarly-named UKCCS case–control study), regional specialist and general cancer registries, Medical Research Council clinical trial registers, and death certificates (Stiller, 2007; Kroll *et al*, 2011). Hence, completeness of ascertainment of diagnosed cases is thought to have been consistently good.

The Carstairs deprivation index is widely used in UK health research (Morgan and Baker, 2006), and has been applied in previous analyses of NRCT data (Committee on Medical Aspects of Radiation in the Environment, 2006; Stiller *et al*, 2008). It has been shown to perform well in comparison with other geographical measures of material disadvantage, such as the closely correlated Townsend index (Morris and Carstairs, 1991). Similar indices, and other measures derived from characteristics of the community in which the child lives, are used in other record-based studies of SES and childhood leukaemia. Interview-based studies may use measures relating to the individual family, such as parental income, education or occupational class (Poole *et al*, 2006). Which type of measure is most appropriate depends on the (unknown) reason for the SES association. A suboptimal choice would be unlikely to reverse an observed association, but might dilute it.

The same method of analysis was used for each of the three study decades. The decades were centred on census years, ensuring that each deprivation index was only applied within 5 years of the census it derived from. Within each decade, wards were grouped into categories representing approximate fifths of the child population, using the deprivation index derived from the corresponding census. Although this approach entailed some loss of information, it enabled comparison of associations between decades, as the raw deprivation scores cannot be directly compared between censuses. The use of quintile categories (the most affluent being the reference group) was consistent with previous studies of childhood leukaemia and SES (Borugian *et al*, 2005; Committee on Medical Aspects of Radiation in the Environment, 2006; Stiller *et al*, 2008). A preliminary analysis found that the observed association between childhood leukaemia and SES did not differ between Wales and the nine Government Office Regions of England in any of the three study decades.

Lymphoid leukaemia cases could not be subclassified, because immunophenotype data were not available consistently throughout the study period. Information on ethnicity was also not available, and no adjustment was made for this, or any other factor that might be associated with SES.

### Interpretation

One possible explanation of the observed association is that leukaemia in children from relatively deprived communities may have been systematically under-recorded. The persistence of the association into the most recent decade suggests that under-diagnosis of leukaemia might be a more important factor than under-registration of diagnosed cases. No appreciable variation in completeness of NRCT registration of leukaemia (or lymphoid leukaemia) with SES was found in a study of cases diagnosed during 2003–2004 (Kroll *et al*, 2011). Improvement over time, and hence attenuation of the association, would have been expected if earlier registration had been seriously incomplete. On the other hand, health service inequalities undoubtedly persist in modern Britain, and children with leukaemia living in poorer communities might be more likely to die from pre-emptive infection without being referred for investigation and diagnosis of leukaemia. Bone marrow examination is not routinely performed in children with severe infection, and the threshold for suspicion of leukaemia might vary with community SES. It is not clear why under-diagnosis (or under-registration) should appear to affect lymphoid leukaemia more than acute myeloid leukaemia, however.

Two well-known hypotheses relate the risk of childhood leukaemia to exposure to infection. Kinlen's ‘population mixing’ hypothesis suggests that new contact between people from different geographical areas increases the risk of childhood leukaemia through increased exposure to unfamiliar infection (Kinlen, 2011). Greaves’ ‘delayed infection’ hypothesis suggests that exposure to common infectious agents can trigger B-precursor lymphoid leukaemia in predisposed children, and that the predisposition is more likely to occur in children who were relatively sheltered from infection in infancy (Greaves, 2006). Either or both of these hypotheses might explain the observed association with affluence, on the assumption of greater population mobility, and/or greater protection of infants from infection, in more affluent communities. The apparent specificity of the association to lymphoid leukaemia is consistent with Greaves’ hypothesis. However, many other hypothesised risk factors for childhood leukaemia might also be associated with affluence.

In conclusion, this study demonstrates the persistence over three decades of higher reported incidence of childhood leukaemia in relatively affluent areas of England and Wales.

## Figures and Tables

**Table 1:** Number of cases by diagnostic group, age group and decade of diagnosis

**Diagnostic group**		**Age at diagnosis (years)**				
**Decade**	**ICCC3**	**<1**	**1–4**	**5–9**	**10–14**	**Total**
						
*Lymphoid leukaemia*	I a					
1996–2005		122	1720	987	593	3422
1986–1995		123	1666	864	503	3156
1976–1985		89	1451	883	587	3010
						
*Acute myeloid leukaemia*	I b					
1996–2005		97	211	162	184	654
1986–1995		82	210	134	188	614
1976–1985		63	178	146	213	600
						
*Chronic myeloproliferative diseases*	I c					
1996–2005		0	8	17	43	68
1986–1995		0	13	20	22	55
1976–1985		3	10	12	27	52
						
*Other myeloproliferative diseases*	I d					
1996–2005		29	39	10	2	80
1986–1995		7	27	2	0	36
1976–1985		4	20	1	0	25
						
*Unspecified and other specified leukaemia*	I e					
1996–2005		11	17	9	9	46
1986–1995		17	15	10	19	61
1976–1985		18	15	10	18	61
						
*Total leukaemia*	I					
1996–2005		259	1995	1185	831	4270
1986–1995		229	1931	1030	732	3922
1976–1985		177	1674	1052	845	3748

Abbreviation: ICCC3=International Classification of Childhood Cancer (3rd edition) excluding disease formerly classified as non-malignant. Childhood leukaemia, England and Wales 1976–2005.

**Table 2:** Rate ratio (RR) by deprivation category relative to the most affluent, adjusted for age group (<1, 1–4, 5–9, 10–14 years), with 95% confidence interval

**Leukaemia group**	**Decade of diagnosis**			
**Deprivation**	**1996–2005**	**1986–1995**	**1976–1985**	**Overall^a^**
*Lymphoid*				
1=affluent	1.00	1.00	1.00	1.00
2	1.00 (0.90–1.10)	1.01 (0.90–1.12)	1.09 (0.98–1.22)	1.03 (0.97–1.09)
3	0.92 (0.83–1.02)	0.95 (0.85–1.06)	0.94 (0.84–1.06)	0.94 (0.88–0.997)
4	0.90 (0.81–0.997)	0.87 (0.77–0.97)	0.92 (0.82–1.03)	0.89 (0.84–0.95)
5=deprived	0.90 (0.81–0.997)	0.88 (0.79–0.98)	0.88 (0.78–0.98)	0.89 (0.83–0.94)
*P*(trend)	0.008	0.001	<0.001	<0.001
RR per category	0.97 (0.95–0.99)	0.96 (0.94–0.98)	0.96 (0.93–0.98)	0.96 (0.95–0.98)
				
*Acute myeloid*				
1=affluent	1.00	1.00	1.00	1.00
2	1.19 (0.94–1.51)	1.07 (0.83–1.39)	0.99 (0.78–1.27)	1.09 (0.94–1.25)
3	0.98 (0.77–1.26)	1.23 (0.95–1.58)	0.90 (0.70–1.16)	1.03 (0.89–1.19)
4	0.96 (0.75–1.23)	1.12 (0.87–1.45)	0.88 (0.69–1.14)	0.98 (0.85–1.13)
5=deprived	1.00 (0.78–1.27)	1.08 (0.84–1.40)	0.94 (0.73–1.20)	1.00 (0.86–1.16)
*P*(trend)	0.397	0.502	0.382	0.520
RR per category	0.98 (0.93–1.03)	1.02 (0.96–1.08)	0.98 (0.92–1.03)	0.99 (0.96–1.02)
				
*Other/unspecified*				
1=affluent	1.00	1.00	1.00	1.00
2	0.99 (0.62–1.59)	0.90 (0.55–1.46)	0.92 (0.54–1.55)	0.94 (0.70–1.25)
3	1.36 (0.87–2.11)	0.69 (0.41–1.16)	0.84 (0.49–1.44)	0.97 (0.73–1.28)
4	1.26 (0.81–1.98)	0.85 (0.52–1.39)	0.90 (0.53–1.53)	1.01 (0.76–1.33)
5=deprived	0.99 (0.62–1.59)	0.93 (0.58–1.51)	0.99 (0.59–1.65)	0.97 (0.73–1.28)
*P*(trend)	0.674	0.727	0.960	0.970
RR per category	1.02 (0.92–1.13)	0.98 (0.88–1.10)	1.00 (0.89–1.12)	1.00 (0.94–1.07)
				
*Total leukaemia*				
1=affluent	1.00	1.00	1.00	1.00
2	1.02 (0.93–1.12)	1.01 (0.92–1.11)	1.07 (0.97–1.18)	1.03 (0.98–1.09)
3	0.94 (0.86–1.04)	0.98 (0.89–1.08)	0.93 (0.84–1.03)	0.95 (0.90–1.01)
4	0.92 (0.84–1.01)	0.90 (0.81–0.99)	0.91 (0.82–1.01)	0.91 (0.86–0.96)
5=deprived	0.91 (0.83–1.01)	0.91 (0.82–1.01)	0.89 (0.80–0.99)	0.91 (0.86–0.96)
*P*(trend)	0.009	0.008	<0.001	<0.001
RR per category	0.97 (0.95–0.99)	0.97 (0.95–0.99)	0.96 (0.94–0.98)	0.97 (0.96–0.98)

Childhood leukaemia, England and Wales 1976–2005.

a Adjusted for age group and decade.
